# The potential correlations between cell-free extracts from *Rhodobacter sphaeroides* grown under low-oxygen conditions and volatile organic compounds in Chinese-style sausage

**DOI:** 10.1016/j.fochx.2024.101967

**Published:** 2024-11-07

**Authors:** Xin Nie, Jingjing Luo, Hongfan Chen, Haomou Pu, Qiqi Luo, Xinhui Wang, Xiaoping Yu, Dayu Liu, Zhiping Zhao

**Affiliations:** a Culinary Science Key Laboratory of Sichuan Provincial Universities, College of Culinary and Food Science Engineering, Sichuan Tourism University, Chengdu 610100, China.; b Meat Processing Key Laboratory of Sichuan Province, College of Food and Biological Engineering, Chengdu Univeristy, Chengdu 610106, China.; c School of Preclinical Medicine, Chengdu University, Chengdu 610106, China.

**Keywords:** Sausage, *Rhodobacter sphaeroides*, Flavor formation, GC–MS, Proteomics

## Abstract

Limited research has explored the use of *Rhodobacter sphaeroides* cell-free extracts (RCFE) in meat processing. To examine the potential application of RCFE in improving the flavor quality of Chinese-style sausage, in this study, we investigated the effects and mechanisms of RCFE grown under low-oxygen conditions on the flavor development of Chinese-style sausage, using GC–MS and 4D label-free proteomics. The GC–MS analysis detected 60 volatile organic compounds, with significant increases in acids, esters, and alcohols following the addition of RCFE (*p* < 0.01). Fifteen differential flavor compounds were identified as potential biomarkers to distinguish sausages. From a total of 2689 proteins, 364 differentially expressed proteins were identified (*p* < 0.05, |Log_2_FC| > 1, and VIP > 1,) in RCFE grown under low- and high-oxygen conditions. KEGG pathway analysis suggested that the RCFE grown under low-oxygen conditions may enhance alcohol and acid levels by upregulating the expression of related enzymes, which subsequently increases ester levels in the sausage.

## Highlights

• *R. sphaeroides* cell-free extracts could significantly increase the acids, esters, and alcohols contents in sausage.
• *R. sphaeroides* cell-free extracts possibly enhanced alcohol and acid levels, and thus increased ester levels in sausage.
• Potential correlation between differential proteins of *R. sphaeroides* and sausage flavor formation was investigated.

## 1 Introduction

*Rhodobacter sphaeroides* (*R. sphaeroides*) is a widely distributed purple photosynthetic bacterium with diverse metabolic capabilities (Tani et al., 2022). It can grow photoautotrophically (CO_2_/light/anoxic), photoheterotrophically (organic compounds/light/anoxic), chemolithotrophically (CO_2_ + H_2_/dark/oxic), and through either respiration (dark/oxic) or fermentation (dark/anoxic) of organic compounds (Mackenzie et al., 2001). As one of the most intensively studied purple phototrophs, *R. sphaeroides* serves as a model organism for fundamental research in photochemistry, metabolism, and regulatory mechanisms (Pitsawong et al., 2023). Additionally, it is capable of biosynthesizing a variety of bioactive compounds such as carotenoids, coenzyme Q_10_, and isoprenoids (Chen et al., 2019).

Traditional Chinese fermented meat products are primarily produced through spontaneous fermentation, where endogenous enzymes and microorganisms hydrolyze carbohydrates, lipids, and proteins into monosaccharides, free fatty acids (FFA), and free amino acids (FAA), which serve as flavor precursors (Ashaolu et al., 2023). Microbial fermentation of carbohydrates produces a variety of flavor compounds, including acids, aldehydes, alcohols, and esters. Pyruvic acid, a key intermediate in carbohydrate catabolism, is converted into compounds such as acetaldehyde, acetic acid, ethanol, and lactic acid through the action of different catalytic enzymes (Lo et al., 2018). FAAs, through processes such as transamination, deamination, and decarboxylation catalyzed by microbial enzymes, form branched-chain alcohols, aldehydes, and organic acids. For instance, amino acids like isoleucine, leucine, valine, and phenylalanine are metabolized by microorganisms into branched-chain aldehydes and alcohols, including 2-methylbutyraldehyde, 3-methylbutyraldehyde, 3-methylbutanol, benzaldehyde, and benzyl alcohol (Olivares et al., 2009). FFAs are oxidized to form alcohols, aldehydes, acids, and alkanes, bringing unique aromas to meat products. Engelvin et al. found that thioesterase produced by *Staphylococcus meatus* promotes FFA deacylation and enhances β-oxidation of fats, leading to the formation of flavor compounds such as valeraldehyde and octanal, which intensify the smoky aroma of sausages (Engelvin et al., 2000). However, during processing and storage, lipids and proteins are prone to excessive oxidation, resulting in undesirable flavors, such as rancidity and spoilage (Neethling et al., 2016).

Metabolites in *R. sphaeroides* cell-free extracts (RCFE) grown under low-oxygen conditions, including carotenoids and coenzyme Q_10_, exhibit functional activities (e.g., anti-cancer and anti-cardiovascular effects) and potent antioxidant properties, potentially inhibiting lipid and protein oxidation in sausages. Furthermore, RCFE are rich in bioactive enzymes (Sasaki et al., 2005), which may enhance flavor formation in meat products. Despite the promising potential, research on the use of RCFE to improve sausage flavor and elucidate the underlying mechanisms remains limited. To examine the potential application of RCFE in improving the flavor quality of Chinese-style sausage, this study explores the effects of RCFE on the flavor of Chinese-style sausage using GC–MS and investigates the mechanisms involved through 4D label-free proteomics, offering new insights into enhancing the flavor quality of traditional Chinese meat products via microbial metabolism.

## 2 Materials and methods

### 2.1 Bacterial growth condition

The growth conditions were performed as described in our previous study (Nie et al., 2021). For low-oxgen growth, the *R. sphaeroides* strain was cultivated in a 1000 mL flask containing 800 mL of malate minimal medium, with continuous shaking at 150 rpm at 32 °C until an OD_600_ of 0.5 was reached. For high-oxygen growth, the strain was cultured in a 1000 mL flask with 250 mL of malate minimal medium, shaking at 180 rpm at 32 °C until an OD_600_ of 0.5 was reached.

### 2.2 Preparation of cell-free extracts from *R. sphaeroides*

Cell cultures were harvested by centrifugation (Xiangyi GL-21 M) at 4500 rpm (Xiangyi, Changsha, China) for 20 min at 4 °C. The resulting cell pellets were resuspended in normal saline and disrupted by ultra-sonication (Scientz-650E, Ningbo Scientz Biotechnology CO., LTD) for 30 min on ice. The cell-free extracts were obtained by centrifugation at 12,000 rpm (TGL-20 M, Xiangyi) for 20 min at 4 °C.

### 2.3 Preparation of Chinese-style sausage

The raw materials consisted of 70 % lean pork and 30 % pork fat, both purchased from Sichuan Goldkinn Foods Company (Suining, China). Salt (25 g) was added per kilogram of raw pork. The control and sample sausage was respectively prepared without and with RCFE, designated as samples C and A, respectively. Cell-free extracts were prepared from 2 L *R. sphaeroides* grown under low-oxygen conditions. To better evaluate the antioxidant activity of the RCFE, no spices or other food additives with antioxidant properties were used in any of the sausages.

After mincing, blending and curing (4 °C, 12 h), the mixtures were stuffed into pig small intestine casings and air-dried for 7 days in a fermenting chamber (BFJX-500, China Expro Co., LTD). The chamber was maintained at 12 °C during the day-time (11.5 h) and 8 °C at night-time (12.5 h), with relative humidity between 70 and 80 % and a wind speed of 0.8–1.2 m/s. The control sausages air-dried for 0 and 7 d termed C0 and C7, respectively. While, the sample sausages air-dried for 0 and 7 d termed A0 and A7, respectively.

### 2.4 Analysis of VOCs in sausage

The VOCs in the Chinese-style sausages were analyzed using solid-phase microextraction gas chromatography–mass spectrometry (SPME-GC–MS, Agilent 5977 A-7890B). A 50/30 μm divinylbenzene/carboxen/polydimethylsiloxane (DVD/CAR/PDMS) fiber was used for VOC extraction from both control and sample sausages. Three grams of minced sausage were transferred into a 40 mL headspace flask, and 1 μL of 2,4,6-trimethyl-pyridine solution was added as an internal standard. The samples were equilibrated at 50 °C for 30 min, followed by extraction with the SPME fiber for 30 min at the same temperature. The fiber was then inserted into the GC injection port for analyte desorption for 5 min at 250 °C. All sausages samples were extracted in triplicate.

Volatile compounds were separated using a Capillary column (HP-5MS UI, 30 m **×** 0.25 mm, film thickness 0.25 μm) with helium as the carrier gas at a flow rate of 1.0 mL/min. The temperature program was as follows: an initial temperature of 40 °C for 1 min, then increased to 85 °C at 3 °C/min for 3 min; then to 105 °C at 2 °C/min for 2 min, followed by 165 °C at 12 °C/min, and finally to 230 °C at 10 °C/min. The MS operating conditions were set as follows: ionization energy of 70 eV; ion source temperature at 230 °C; quadrupole temperature at 150 °C; detector voltage at 350 V; a scan range of 40–550 *m/z*. Volatile compounds were identified by comparing mass spectra data of the sausage samples with those in the NISTDEMO 14 L library, considering only compounds with a matching probability above 80 %.

### 2.5 Total protein extraction from *R. sphaeroides*

Frozen samples were placed on ice, and an appropriate volume of protein lysis buffer (8 M urea, 1 % SDS) containing protease inhibitors was added to inhibit protease activity. The mixture was sonicated for 2 min at a low temperature, followed by incubation for 30 min to ensure complete lysis. After centrifugation at 12,000 *g* for 30 min at 4 °C, the protein concentration in the supernatant was determined using the Bicinchoninic Acid (BCA) Protein Assay Kit (Thermo Scientific, Rockford, IL, USA) following the manufacturer's instructions.

### 2.6 Lysis treatment of total protein from *R. sphaeroides*

A 100 μg aliquot of protein sample was mixed with triethylammonium bicarbonate (TEAB) buffer to a final concentration of 100 mM. Tris (2-carboxyethyl)phosphine (TCEP) was added to a final concentration of 10 mM, and the reaction was incubated at 37 °C for 60 min. Subsequently, iodoacetamide (IAM) was added to a final concentration of 40 mM, and the mixture was incubated for 40 min at room temperature in the dark. Pre-chilled acetone (6:1 *v*/v, acetone) was added to the samples, and the mixtures were left to precipitate for 4 h at −20 °C. After centrifugation at 10,000 *g* for 20 min, the pellet was collected and dissolved in 100 μL of 100 mM TEAB solution. The proteins were then digested with trypsin at a 1:50 trypsin-to-protein ratio overnight at 37 °C.

The resulting peptides were vacuum-dried and resuspended in 0.1 % trifluoroacetic acid (TFA). Samples were desalted using HLB and vacuum-dried again. Peptide concentrations were measured using a peptide quantification kit (Thermo Scientific, Rockford, IL, USA). A loading buffer was added to each tube to prepare the samples for mass spectrometry analysis, with a final peptide concentration of 0.25 μg/μL per sample.

### 2.7 LC-MS/MS analysis

Trypsin-digested peptides were analyzed using an EASY nLC-1200 system (Thermo Scientific, Rockford, IL, USA) coupled with a timsTOF Pro2 mass spectrometer (Bruker Daltonik GmbH, Germany) at Majorbio Bio-Pharm Technology Co. Ltd. (Shanghai, China). A C18-reversed phase column (75 μm × 25 cm, Ionopticks, USA) was equilibrated with solvent A (A:2 % ACN with 0.1 % formic acid) and solvent B (B: 80 % ACN with 0.1 % formic acid). The peptides were eluted using the following gradient: 0–45 min, 3 %–28 % B; 45–50 min, 28 %–44 % B; 50–55 min, 44 %–90 % B; 55–60 min, 90 %–90 % B. The tryptic peptides were separated at a flow rate of 250 nL/min. Peptides were separated by an ultrahigh performance liquid phase system and subjected to a capillary ion source before analysis by the timsTOF Pro2 (Bruker Daltonik GmbH, Germany), operating with an electrospray voltage of 1.5 kV. Peptide parent ions and their secondary fragments were detected and analyzed using high-resolution TOF, with a secondary MS scan range of 100–1700 *m*/*z*. Data acquisition was performed using the parallel accumulation serial fragmentation (PASEF) mode. The second MS stage, capturing parent ions with charge states of 0–5, was recorded using 10 PASEF scans, with a dynamic exclusion time of 24 s for MS/MS scans.

### 2.8 Qualitative and quantitative analysis of protein

MS/MS spectra were analyzed using MaxQuant version 2.0.3.1. Peptides were identified based on their highest matching score with the predicted mass in the database, allowing the identification of parent proteins. Search parameters were set to include tryptic digestion with up to two missed cleavages, carbamidomethylation of cysteines as a fixed modification, and oxidation of methionines and protein N-terminal acetylation as variable modifications. The false discovery rate (FDR) for peptide identification was set at ≤0.01. Protein identification required a minimum of one unique peptide match.

### 2.9 Data analysis

Data processing was conducted using Microsoft Office 2021 (Microsoft, Inc., Redmond, WA, USA). Statistical analysis, including significance (*P* values) and fold changes (FC) between groups, was performed using SPSS 27 (IBM, Inc., Armonk, NY, USA). Principal component analysis (PCA) and partial least squares discriminant analysis (PLS-DA) were carried out using SIMCA (version 14.1, Umetrics, Umea, Sweden). Gene Ontology (GO) annotation and functional clustering of differential proteins were performed using the GO database (Gene Ontology, http://geneontology.org/), focusing on biological processes, cellular components, and molecular functions. KEGG pathway analysis (Kyoto Encyclopedia of Genes and Genomes, http://www.genome.jp/kegg/) was used to explore metabolic pathways associated with the differential proteins. Visualization, including heat maps, clustering dendrograms, volcano plots, and bar charts, were created using R (version 4.2.3, R Foundation for Statistical Computing, Vienna, Austria).

## 3 Results and discussion

### 3.1 Analysis of VOCs in sausages

**Fig. 1:**
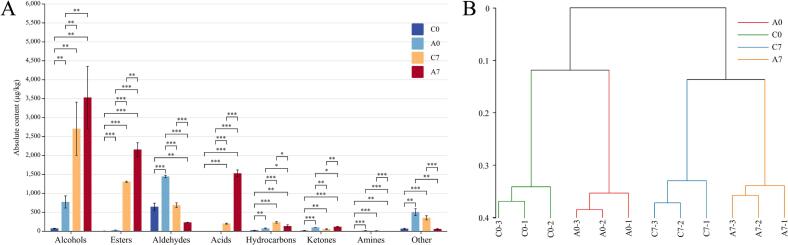
Content of volatile flavor compounds (A) and volatile flavor clustering analysis in different sausages (B).

A total of 60 volatile organic compounds (VOCs) were detected in the four sausage samples (C0 and C7, A0 and A7) using GC–MS. These included 13 alcohols, 10 esters, 7 aldehydes, 4 acids, 13 hydrocarbons, 3 ketones, 3 amines, and 7 other compounds. The VOC composition for each sausage group is shown in Fig. 1 A. The C0, C7, A0, and A7 samples contained 22, 32, 30, and 28 VOCs, respectively, with total concentrations of 834.43 μg/kg, 5559.84 μg/kg, 2942.20 μg/kg, and 7732.87 μg/kg. Upon the addition of RCFE, there were significant changes in VOC content, particularly in alcohols, esters, acids, hydrocarbons, and ketones, which increased significantly (*p* < 0.05), indicating that these cell-free extracts effectively enrich the flavor profile of Chinese-style sausages. In contrast, A7 samples showed a significant decrease in aldehydes and other compounds compared to C7 (*p* < 0.05), which may be due to the inhibition of lipid oxidation by bioactive components in the RCFE.

Alcohols were the most abundant VOCs, primarily derived from various pathways including carbohydrate fermentation, methyl ketone reduction, amino acid metabolism, and lipid oxidation (Sidira et al., 2015). While saturated alcohols generally have minimal impact on flavor due to their high thresholds, unsaturated alcohols play a more significant role (Bianchi et al., 2007). Six types of unsaturated alcohols were detected: trans-2-octen-1-ol, 4-methyldecan-5-ol, (2Z)-2-octene-1-ol, 1-octene-3-ol, phenethyl alcohol, 3,5-octadien-2-ol, 1-heptyn-3-ol, and linalool. Among these, trans-2-octen-1-ol and 1-octene-3-ol are primarily formed from the oxidative cleavage of linoleic acid. 1-Octene-3-ol, which has a strong mushroom aroma, is produced through the oxidation of linoleic acid (Grilo & Wang, 2021). Under the catalytic action of lipoxygenase, linoleic acid forms 10(*R*)-ROOH, which is then cleaved by hydroperoxide lyase (HPL) to yield 1-octene-3-ol. (2Z)-2-octene-1-ol, responsible for a rancid odor, is primarily derived from the oxidation of arachidonic acid. Arachidonic acid, catalyzed by arachidonate lipoxygenase (ALOX), forms various hydroperoxides, among which 12-ROOH is further degraded by hydroperoxide lyase (HPL) to form (2Z)-2-octene-1-ol (Giri et al., 2010). In this study, the experimental group exhibited higher levels of trans-2-octen-1-ol and 1-octene-3-ol, along with lower levels of (2Z)-2-octene-1-ol compared to the control group. These findings indicate that the addition of RCFE enhances the mushroom aroma while reducing the rancid oil odor in sausages.

Ester compounds are mainly formed through the esterification of alcohols and acids, facilitated by both non-enzymatic and enzymatic catalysis, often driven by microbial activity. In this study, the addition of RCFE led to a significant increase in ester content (*p* < 0.01). Esters derived from short-chain fatty acids typically impart a fruity aroma, while those from long-chain fatty acids contribute to an oily flavor profile. Due to their low sensory thresholds, esters play a pivotal role in the flavor development of meat products. The experimental group exhibited significantly higher ester content than that of the control group (*p* < 0.05), likely because the RCFE accelerated the production of alcohols and acids during sausage fermentation, consistent with the findings of Paronyan et al. (Paronyan, 2002) and Zagrodnik et al. (Zagrodnik & Łaniecki, 2017). Ethyl esters of fatty acids were the predominant ester compounds detected, formed through microbial esterase activity or chemical esterification (Nykänen, 1986). These esters are key contributors to the flavor of meat products. Ethyl 3-methylbutyrate, ethyl 2-methylbutyrate, and ethyl 2-methylpropionate were only detected on day 7, with significantly higher concentrations in the experimental group compared to the control (*p* < 0.05). This may be due to the higher initial alcohol content in the sausages resulting from the addition of RCFE. Hexyl formate, with its apple-like sweet aroma, was the most abundant ester, and was also only detected on day 7 of drying, making it an important component of the characteristic sausage flavor. Esters generally contribute sweet, floral, and fruity aromas, such as ethyl 2-methylbutyrate (with a fruity scent) and isoamyl formate (with plum and ether-like odors). The substantial increase in ester content following the addition of RCFE (*p* < 0.01) underscores their significant role in enhancing the flavor profile of sausages.

Aldehydes primarily arise from the oxidation of unsaturated fatty acids, such as oleic acid and linoleic acid, and the degradation of amino acids. These compounds have low thresholds (Wang et al., 2016). In this study, seven aldehydes were identified: heptanal, 10-decanal, nonanal, 2-indecent, (E)-2-octenal, (2E,4E)-2,4-nonradial, and hexanal. Hexanal and heptanal are important oxidative cleavage products of linoleic acid (Varlet et al., 2007). Linoleic acid can undergo both auto-oxidation and enzymatic oxidation to form 13(*S*)-ROOH, which is then degraded by HPL to yield hexanal and heptanal (Wu et al., 2021). Hexanal, the most abundant aldehyde in sausages, is a major contributor to undesirable fatty flavors (Bis-Souza et al., 2019). In this study, the addition of RCFE significantly reduced hexanal content, suggesting that these extracts mitigate off-flavors in sausages, possibly by inhibiting linoleic acid oxidation, which was similar to the findings reported by Rajamani et al. (Rajamani et al., 2018).

Acid compounds are essential precursors for ester formation and play a significant role in flavor development in sausages. Four acids were detected in this study: 3-methylvaleric acid, 2-methylbutyric acid, isovaleric acid, and isobutyric acid. These acids were only present on day 7 of drying, indicating that the RCFE did not affect the original acid composition of the sausages. However, their levels significantly increased following the addition of RCFE, suggesting these cell-free extracts promoted acid production, thereby enhancing ester content, consistent with the findings of Paronyan et al. (Paronyan, 2002). The notable increase in isovaleric acid and isobutyric acid may result from pyruvic acid metabolism, where acetic acid combines with propionyl-CoA and acetyl-CoA to form these acids under specific conditions (Spirito et al., 2014).

To further investigate differences in volatile flavors among the sausage groups, a hierarchical clustering analysis was performed, as shown in Fig. 1B. The analysis grouped the four sausage samples into two clusters: C0 and A0 in one, and C7 and A7 in the other.

### 3.2 Multivariate statistical analysis of volatile flavor of different sausages

**Fig. 2:**
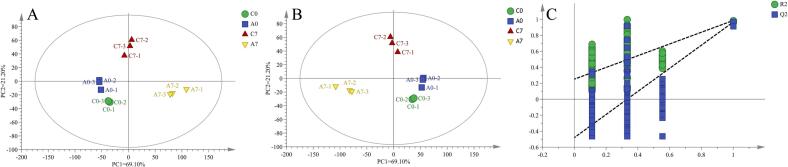
PCA score scatter plot of volatile flavors in different sausages (A), PLS-DA score scatter plot (B), and PLS-DA model permutation test results (C).

PCA results are shown in Fig. 2A. The contributions of PC 1 and PC 2 were 69.10 % and 21.20 %, respectively, indicating that the PCA model effectively captures the overall flavor profiles of the sausage samples. In the PLS-DA analysis (Fig. 2B), sausage samples from different groups were positioned in distinct regions of the coordinate system with no overlap, demonstrating significant flavor differences among the groups (Worley & Powers, 2013). Specifically, the A0 and C0 samples were located on the right side of the axis, while the A7 and C7 samples were on the left, indicating similar flavor characteristics within A0 and C0, and within A7 and C7, consistent with the results of the hierarchical clustering analysis and PCA. A permutation test with 200 iterations (Fig. 2C) confirmed the stability and reliability of the PLS-DA model, with no indication of overfitting.

### 3.3 Selection of differential VOCs

**Fig. 3:**
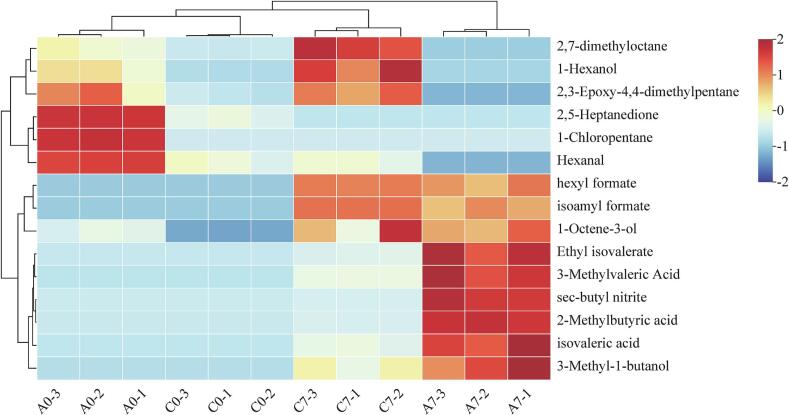
Hierarchical clustering analysis of differential flavor compounds in four types of sausages.

Differential VOCs (Table 1) among the four sausages (C0 and C7, A0 and A7) were screened based on their Variable Importance in Projection (VIP) scores from the PLS-DA analysis. Fifteen differential VOCs (VIP > 1) were identified, which can serve as potential biomarkers for distinguishing sausages with varying storage times. These compounds include 1-hexanol, hexanal, 3-methyl-1-butanol, hexyl formate, 1-chloropentane, 2-methylbutyric acid, 2,3-epoxy-4,4-dimethylpentane, 1-octene-3-ol, isoamyl formate, ethyl isovalerate, 3-methylvaleric acid, 2,5-heptanedione, isovaleric acid, sec-butyl nitrite, and 2,7-dimethyloctane. To visually present the differences in content of these differential VOCs, clustering was performed and displayed as a heatmap (Fig. 3). After the addition of RCFE, sausage samples exhibited higher levels of hexanal, 1-chloropentane, and 2,5-heptanedione. By the end of the drying process, control group (C7) sausage had higher levels of 1-hexanol, 2,3-epoxy-4,4-dimethylpentane, and 2,7-dimethyloctane, whereas the experimental group (A7) sausage contained elevated levels of 3-methyl-1-butanol, hexyl formate, 2-methylbutyric acid, 1-octene-3-ol, isoamyl formate, ethyl isovalerate, 3-methylvaleric acid, isovaleric acid, and sec-butyl nitrite. Among these, hexyl formate, isoamyl formate, and ethyl isovalerate have fruity aromas (Liu et al., 2021), while 1-octene-3-ol and 3-methylvaleric acid contribute mushroom and herbal aromas, respectively (Hou et al., 2024). The significant increase in these flavor compounds following the addition of RCFE highlights their role in enhancing the flavor profile of the sausages.

**Table 1:** VIP values of differential flavor compounds and their aroma characteristics.

Volatile flavor components	RT	CAS	VIP	Aroma description
1-Hexanol	6.333	111–273	3.04733	The smell of grass, leaves
Hexanal	4.392	66–25-1	3.01892	Grass, rancidity
3-Methyl-1-butanol	3.192	123–51-3	2.80046	Bitter almond
Hexyl formate	6.386	629–33-4	2.33896	Apple sweetness
1-Chloropentane	3.757	543–59-9	2.10448	
2-Methylbutyric acid	6.292	116–53-0	1.5588	Cheesy, sweaty
2,3-Epoxy-4,4-dimethyl pentane	3.751	53,897–30-6	1.47954	
1-Octene-3-ol	10.501	3391-86-4	1.47119	Mushroom aroma
Isoamyl formate	3.763	110–45-2	1.43513	Plum and ether like aroma
Ethyl isovalerate	5.751	108–64-5	1.36983	Fruity aroma
3-Methylvaleric Acid	6.025	105–43-1	1.22358	Herb aroma
2,5-Heptanedione	10.629	1703-51-1	1.21615	
Isovaleric acid	5.989	503–74-2	1.18788	Sharp, sour taste
sec-Butyl nitrite	3.617	924–43-6	1.15507	
2,7-Dmethyloctane	10.612	1072-16-8	1.00548

### 3.4 Protein analysis in *R. sphaeroides* grown under higher and lower oxygen conditions

**Fig. 4:**
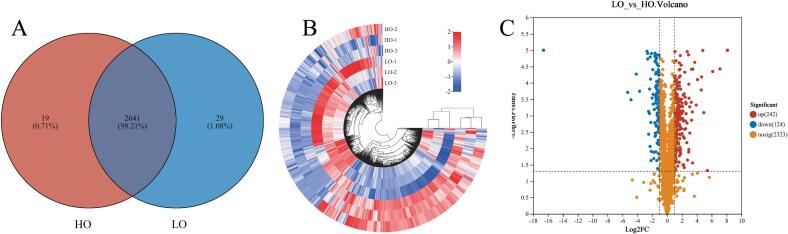
Impact of low-oxygen and high-oxygen cultures on the protein composition of *R. sphaeroides* (A: Venn analysis of common proteins; B: Heatmap clustering analysis of total proteins; C: Analysis of differentially expressed proteins).

Protein changes in *R. sphaeroides* grown under different oxygen conditions were analyzed using 4D lable-free proteomics, as shown in Fig. 4. After excluding proteins with missing values greater than 90 %, a total of 2689 proteins were identified. Of these, 2660 were detected in the high-oxygen (HO) group and 2670 in the low-oxygen (LO) group, with 2641 proteins shared between both groups (Fig. 4A). A heatmap (Fig. 4B) revealed distinct clustering patterns of the 2689 identified proteins between the low-oxygen and high-oxygen cultures, indicating significant differences in protein composition. Samples from each culture condition clustered into separate groups, demonstrating strong reproducibility within groups and clear differentiation between the HO and LO conditions. A volcano plot (Fig. 4C) was used to identify proteins with significant changes in abundance, based on the criteria of |Log_2_FC| > 1 and *p* < 0.05 (Mi et al., 2019). The low-oxygen culture led to significant changes in the abundance of 366 proteins, with 242 upregulated and 124 downregulated. The increase in specific protein levels under low-oxygen conditions in RCFE may influence the flavor profile of sausages.

### 3.5 Multivariate statistical analysis of *R. sphaeroides* proteins

**Fig. 5:**
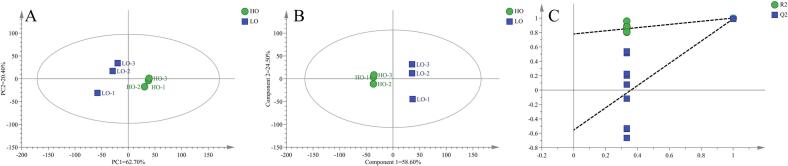
PCA score plot (A), OPLS-DA score plot (B), and permutation test results (C) for the proteomics of *R. sphaeroides* cultured under low-oxygen and high-oxygen conditions.

Principal Component Analysis (PCA) of *R. sphaeroides* protein composition is shown in Fig. 5A. The model explained 81.10 % of the variance, indicating that the PCA effectively captured the differences in protein composition across samples. Orthogonal Partial Least Squares Discriminant Analysis (OPLS-DA) results are displayed in Fig. 5B. Samples from the low-oxygen and high-oxygen cultures were clearly separated into distinct regions of the coordinate system, with no overlap, confirming significant differences in protein composition between the two groups (HO and LO). These findings are consistent with the total protein clustering analysis. To evaluate the stability of the OPLS-DA model, a permutation test with 200 iterations was conducted, as shown in Fig. 5C. The Q2 regression line intersected the Y-axis in the negative region, indicating that the OPLS-DA model was stable and reliable.

### 3.6 Screening and bioinformatics analysis of differential proteins in *R. sphaeroides* grown under low- and high-oxygen conditions

**Fig. 6:**
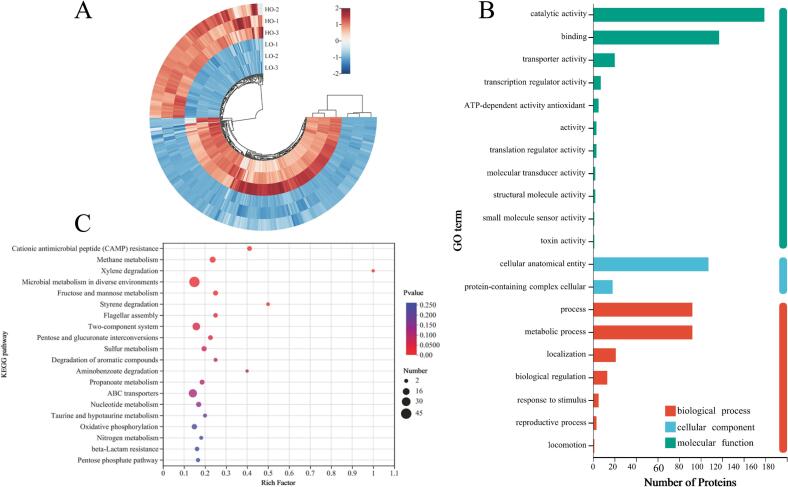
Analysis of diferential proteins in *R. sphaeroides* under low- and high-oxygen conditions (A: clustering heatmap analysis; B: GO annotation analysis; C: KEGG pathway enrichment).

Differentially expressed proteins (DEPs) were selected based on the criteria of *p* < 0.05, |Log_2_FC| > 1, and VIP > 1 (Jia et al., 2021), as summarized in Table S1. A total of 364 DEPs were identified from the 2689 proteins, with 242 upregulated and 122 downregulated in the LO group. A clustering heatmap (Fig. 6A) was generated to visually represent the expression differences between the LO and HO groups.

Gene Ontology (GO) annotation was used to analyze the functional characteristics of the 364 DEPs, as shown in Fig. 6B. These proteins were classified into three main categories: biological processes, molecular functions, and cellular components. In terms of biological processes, the majority of DEPs were involved in cellular processes (92 proteins) and metabolic processes (92 proteins), with others participating in localization (21 proteins), biological regulation (13 proteins), response to stimulus (5 proteins), reproductive processes (3 proteins), and locomotion (1 protein). Molecular function analysis revealed that most DEPs exhibited catalytic activity (159 proteins) and binding functions (117 proteins), followed by transporter activity (20 proteins) and transcription regulator activity (7 proteins). The large number of proteins with catalytic activity suggests that cultivation conditions have a significant impact on the catalytic processes of *R. sphaeroides*, consistent with the findings of Pan et al. (Pan et al., 2021). For cellular components, the DEPs were mainly associated with cellular anatomical entities (107 proteins) and protein-containing complexes (18 proteins).

To further investigate the impact of high- and low-oxygen conditions on the metabolic pathways of *R. sphaeroides*, KEGG pathway enrichment analysis was performed on the differential proteins. The results, presented in Fig. 6C, showed that 364 differential proteins were enriched across 82 pathways, with 8 metabolic pathways showing significant enrichment (*p < 0.05*). The most significantly enriched pathway was Cationic Antimicrobial Peptide (CAMP) resistance, involving 7 differential proteins (Q3J0Q1, Q3J0J9, A3PP55, Q3IX10, Q3J1D3, Q3HKN1, and Q3IX80). Methane metabolism was the sencond most significantly enriched pathway, with 13 proteins (Q3IYF0, A0A7Z6QWH9, A0A7Z6QYK9, A0A239DEP3, Q3IYF1, Q53176, B9KPE0, P29271, Q3IYB9, B9KQK9, A0A3G6WJG0, Q3J307, and Q3IXS0) identified. Microbial metabolism in diverse environments had the largest number of enriched proteins, with 45 differential proteins. The significant enrichment of the CAMP resistance suggests that cultivation under different oxygen conditions has a substantial impact on the development of antimicrobial resistance in *R. sphaeroides* (Ye et al., 2021).

Methane metabolism, which converts inorganic carbon sources such as methane into organic compounds like acetic acid, was also affected. In low-oxygen conditions, the expression levels of acetate kinase (Q3IYF1), phosphate acetyltransferase (Q3IYF0), and 3-hydroxybutyryl-CoA dehydratase (A0A239DEP3) were significantly upregulated compared to high-oxygen conditions. Acetate kinase catalyzes the dephosphorylation of acetyl phosphate to produce acetic acid (Kuit et al., 2012), while phosphate acetyltransferase catalyzes the conversion of acetyl groups in acetyl CoA with phosphate to generate acetyl phosphate, which can subsequently be hydrolyzed to produce acetic acid. This increase may contribute to the enhanced formation of acidic compounds in sausages.

### 3.7 Potential correlation analysis between differential proteins in RCFE and sausage flavor formation

**Fig. 7:**
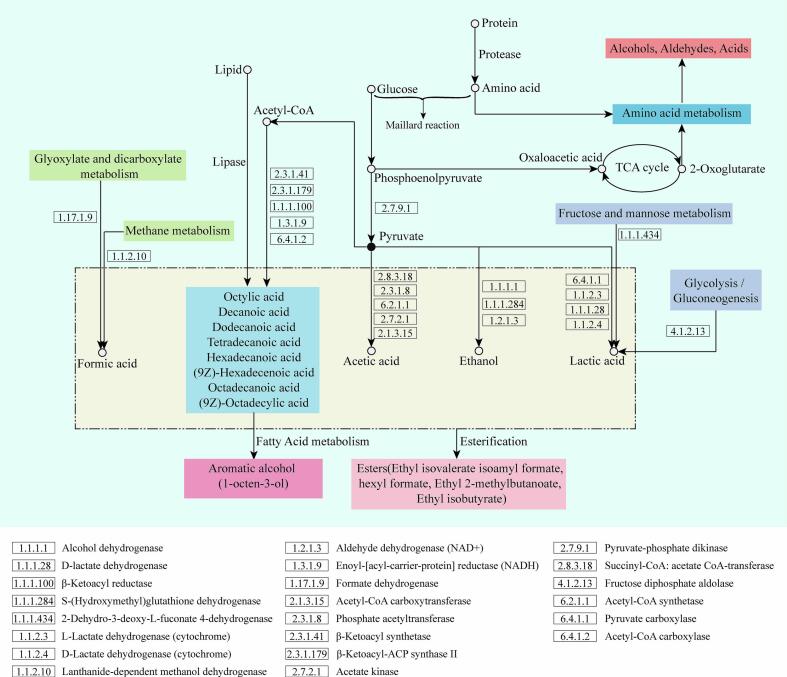
Metabolic pathway of sausage flavor formation.

To further investigate the mechanism by which RCFE influence the volatile flavor of sausages, the 364 differential proteins were analyzed using the KEGG database to identify their associated metabolic pathways and relevant upstream and downstream products. Metabolic pathway diagrams were constructed, as shown in Fig. 7.

In sausages, glucose was first metabolized through glycolysis to form phosphoenolpyruvate, which was subsequently converted into oxaloacetic acid and then into 2-oxoglutarate via TCA cycle (Li et al., 2023). This was further transformed into alcohols, aldehydes, and acids through amino acid metabolism. Concurrently, phosphoenolpyruvate was converted into pyruvate, regulated by pyruvate-phosphate dikinase, and was further metabolized into acetic acid, lactic acid, and ethanol through various catalytic pathways. These compounds, along with formic acid produced via methane metabolism and glyoxylate and dicarboxylate metabolism, underwent esterification to generate ethyl and formate esters. Pyruvate is a key source of acetic acid, ethanol, and lactic acid, with different catalytic pathways producing a range of metabolic products. Acetic acid is primarily produced under the catalysis by enzymes succinyl-CoA: acetate CoA-transferase, phosphate acetyltransferase, acetyl-CoA synthetase, acetate kinase, and acetyl-CoA carboxytransferase (Yao et al., 2020).

Adding RCFE grown under low-oxygen conditions significantly increased the levels of bioactive proteins, which promoted the metabolism and synthesis of alcohols and fatty acids in sausages during fermentation, thereby indirectly enhancing ester production and improving flavor. Succinyl-CoA: acetate CoA-transferase, phosphate acetyltransferase, and acetate kinase were all significantly upregulated in the low-oxygen RCFE, suggesting that these extracts contribute to the increased levels of acids such as 3-methylvaleric acid, 2-methylbutyric acid, isovaleric acid, and isobutyric acid in sausages. This may be due to the enzymatic systems in the cell-free extracts catalyzing acid production under specific conditions (Zagrodnik & Łaniecki, 2017).

Ethanol is mainly produced by alcohol dehydrogenase, S-(hydroxymethyl)glutathione dehydrogenase, and aldehyde dehydrogenase (NAD+) (de Smidt et al., 2012), while lactic acid is produced by pyruvate carboxylase, L-lactate dehydrogenase (cytochrome), D-lactate dehydrogenase, and D-lactate dehydrogenase (cytochrome). Lactic acid is also derived from fructose and mannose metabolism, and the glycolysis/gluconeogenesis pathways, catalyzed by 2-dehydro-3-deoxy-L-fuconate 4-dehydrogenase and fructose diphosphate aldolase (Lee et al., 2019).

Free fatty acids, key flavor precursors in meat products, are derived from the hydrolysis of fats and produced through fatty acid biosynthesis pathways. They can be further converted into alcohols, such as 1-octen-3-ol, via fatty acid metabolism. After the addition of low-oxygen RCFE, levels of 1-hexanol, heptan-1-ol, (2Z)-2-octene-1-ol, 3,5-octadien-2-ol, 1-heptyn-3-ol, and 1-octen-3-ol significantly increased, indicating that these extracts help elevate the initial alcohol content in sausages (Costa et al., 2015). The concurrent increase in alcohol and acid levels further enhances ester content, improving the overall sensory quality of the sausages.

## 4 Conclusion

This study investigated the effects and mechanisms of RCFE on the flavor formation of Chinese-style sausages using GC–MS and 4D label-free proteomics. GC–MS analysis identified 60 VOCs across all sausage samples, highlighting significant differences in volatile flavor profiles. The addition of RCFE significantly enhanced the formation of ester compounds, contributing to an overall improvement in sausage flavor. Through VIP value analysis, 15 key differential flavor compounds were identified. The 4D label-free proteomics analysis showed that the protein content in *R. sphaeroides* was significantly influenced by the culture conditions, with 364 differentially expressed proteins identified based on *p* < 0.05, |Log_2_FC| > 1, and VIP > 1. These proteins were primarily associated with biological processes, molecular functions, and cellular components. The RCFE possibly increased the levels of alcohols and acids in sausages, leading to a corresponding rise in ester content. Future studies involving metabolomics and high-throughput sequencing will further explore the metabolic changes in sausages with RCFE, shedding more light on their effects on sausage quality. On the other hand, isolation and identification of the functional components in RCFE will be performed to promote the application of *R. sphaeroides* in food industry. The present study provides new insights into improving the flavor quality of traditional Chinese fermented meat products using *R. sphaeroides*.

## CRediT authorship contribution statement

**Xin Nie:** Writing – original draft, Methodology, Formal analysis, Data curation. **Jingjing Luo:** Writing – original draft, Methodology, Investigation. **Hongfan Chen:** Formal analysis. **Haomou Pu:** Writing – review & editing. **Qiqi Luo:** Data curation. **Xinhui Wang:** Resources. **Xiaoping Yu:** Supervision, Methodology, Formal analysis, Data curation. **Dayu Liu:** Formal analysis. **Zhiping Zhao:** Writing – review & editing, Supervision, Project administration, Funding acquisition, Conceptualization.

## Declaration of competing interest

The authors declare that they have no known competing financial interests or personal relationships that could have appeared to influence the work reported in this paper.

## Data Availability

Data will be made available on request.

## Appendix A Supplementary data

Supplementary material Image 1

## References

[bb0005] Ashaolu T.J., Khalifa I., Mesak M.A., Lorenzo J.M., Farag M.A. (2023). A comprehensive review of the role of microorganisms on texture change, flavor and biogenic amines formation in fermented meat with their action mechanisms and safety. Critical Reviews in Food Science and Nutrition.

[bb0010] Bianchi F., Cantoni C., Careri M., Chiesa L., Musci M., Pinna A. (2007). Characterization of the aromatic profile for the authentication and differentiation of typical Italian dry-sausages. Talanta.

[bb0015] Bis-Souza C.V., Pateiro M., Domínguez R., Lorenzo J.M., Penna A.L.B., da Silva Barretto A.C. (2019). Volatile profile of fermented sausages with commercial probiotic strains and fructooligosaccharides. Journal of Food Science and Technology.

[bb0020] Chen X., Jiang X., Xu M., Zhang M., Huang R., Huang J., Qi F. (2019). Co-production of farnesol and coenzyme Q(10) from metabolically engineered *Rhodobacter sphaeroides*. Microbial Cell Factories.

[bb0025] Costa R.L., Oliveira T.V., Ferreira J.D.S., Cardoso V.L., Batista F.R.X. (2015). Prospective technology on bioethanol production from photofermentation. Bioresource Technology.

[bb0030] Engelvin G., Feron G., Perrin C., Mollé D., Talon R. (2000). Identification of β-oxidation and thioesterase activities in *Staphylococcus carnosus* 833 strain. FEMS Microbiology Letters.

[bb0035] Giri A., Osako K., Ohshima T. (2010). Identification and characterisation of headspace volatiles of fish miso, a Japanese fish meat based fermented paste, with special emphasis on effect of fish species and meat washing. Food Chemistry.

[bb0040] Grilo F.S., Wang S.C. (2021). Walnut (*Juglans regia L*.) volatile compounds indicate kernel and oil oxidation. Foods.

[bb0045] Hou Z., Xia R., Li Y., Xu H., Wang Y., Feng Y., Pan S., Wang Z., Ren H., Qian G., Wang H., Zhu J., Xin G. (2024). Key components, formation pathways, affecting factors, and emerging analytical strategies for edible mushrooms aroma: A review. Food Chemistry.

[bb0050] Jia W., Shi Q., Zhang R., Shi L., Chu X. (2021). Unraveling proteome changes of irradiated goat meat and its relationship to off-flavor analyzed by high-throughput proteomics analysis. Food Chemistry.

[bb0055] Kuit W., Minton N.P., López-Contreras A.M., Eggink G. (2012). Disruption of the acetate kinase (ack) gene of *Clostridium acetobutylicum* results in delayed acetate production. Applied Microbiology and Biotechnology.

[bb0060] Lee J., Yesilkanal A.E., Wynne J.P., Frankenberger C., Liu J., Yan J., Rosner M.R. (2019). Effective breast cancer combination therapy targeting BACH1 and mitochondrial metabolism. Nature.

[bb0065] Li W., Huo J., Berik E., Wu W., Hou J., Long H., Lei M., Li Z., Zhang Z., Wu W. (2023). Determination of the intermediates in glycolysis and tricarboxylic acid cycle with an improved derivatization strategy using gas chromatography-mass spectrometry in complex samples. Journal of Chromatography A.

[bb0070] Liu J., Wan P., Xie C., Chen D.W. (2021). Key aroma-active compounds in brown sugar and their influence on sweetness. Food Chemistry.

[bb0075] Lo R., Ho V.T.T., Bansal N., Turner M.S. (2018). The genetic basis underlying variation in production of the flavour compound diacetyl by *lactobacillus rhamnosus* strains in milk. International Journal of Food Microbiology.

[bb0080] Mackenzie C., Choudhary M., Larimer F.W., Predki P.F., Stilwagen S., Armitage J.P., Kaplan S. (2001). The home stretch, a first analysis of the nearly completed genome of *Rhodobacter sphaeroides* 2.4.1. Photosynthesis Research.

[bb0085] Mi S., Li X., Zhang C., Liu J., Huang D. (2019). Characterization and discrimination of Tibetan and Duroc x (landrace x Yorkshire) pork using label-free quantitative proteomics analysis. Food Research International.

[bb0090] Neethling J., Hoffman L.C., Muller M. (2016). Factors influencing the flavour of game meat: A review. Meat Science.

[bb0095] Nie X., Jäger A., Börner J., Klug G. (2021). Interplay between formation of photosynthetic complexes and expression of genes for iron–sulfur cluster assembly in *Rhodobacter sphaeroides*?. Photosynthesis Research.

[bb0100] Nykänen L. (1986). Formation and occurrence of flavor compounds in wine and distilled alcoholic beverages. American Journal of Enology and Viticulture.

[bb0105] Olivares A., Navarro J.L., Flores M. (2009). Establishment of the contribution of volatile compounds to the aroma of fermented sausages at different stages of processing and storage. Food Chemistry.

[bb0110] Pan C., Ma J., Tao F., Ji C., Zhao Y., Chen S., Yang X. (2021). Novel insight into the antioxidant proteins derived from laver (*Porphyra haitanensis*) by proteomics analysis and protein based bioinformatics. Food Bioscience.

[bb0115] Paronyan A.K. (2002). Consumption of organic carbon sources and biosynthesis of lactic acid by the photosynthetic bacterium *Rhodobactersp*. D-4. Applied Biochemistry and Microbiology.

[bb0120] Pitsawong W., Pádua R.A.P., Grant T., Hoemberger M., Otten R., Bradshaw N., Kern D. (2023). From primordial clocks to circadian oscillators. Nature.

[bb0125] Rajamani K., Balasubramanian T., Thirugnanasambandan S.S. (2018). Bioassay-guided isolation of triterpene from brown alga *Padina boergesenii* possess anti-inflammatory and anti-angiogenic potential with kinetic inhibition of β-carotene linoleate system. LWT.

[bb0130] Sasaki K., Watanabe M., Suda Y., Ishizuka A., Noparatnaraporn N. (2005). Applications of photosynthetic bacteria for medical fields. Journal of Bioscience and Bioengineering.

[bb0135] Sidira M., Kandylis P., Kanellaki M., Kourkoutas Y. (2015). Effect of immobilized *lactobacillus casei* on the evolution of flavor compounds in probiotic dry-fermented sausages during ripening. Meat Science.

[bb0140] de Smidt O., du Preez J.C., Albertyn J. (2012). Molecular and physiological aspects of alcohol dehydrogenases in the ethanol metabolism of *Saccharomyces cerevisiae*. FEMS Yeast Research.

[bb0145] Spirito C.M., Richter H., Rabaey K., Stams A.J.M., Angenent L.T. (2014). Chain elongation in anaerobic reactor microbiomes to recover resources from waste. Current Opinion in Biotechnology.

[bb0150] Tani K., Kanno R., Kikuchi R., Kawamura S., Nagashima K.V.P., Hall M., Wang-Otomo Z.-Y. (2022). Asymmetric structure of the native *Rhodobacter sphaeroides* dimeric LH1–RC complex. Nature Communications.

[bb0155] Varlet V., Prost C., Serot T. (2007). Analytical, nutritional and clinical methods. Food Chemistry.

[bb0160] Wang Y., Jiang Y., Cao J., Chen Y., Sun Y., Zeng X., Pan D., Ou C., Gan N. (2016). Study on lipolysis-oxidation and volatile flavour compounds of dry-cured goose with different curing salt content during production. Food Chemistry.

[bb0165] Worley B., Powers R. (2013). Multivariate analysis in metabolomics. Current Metabolomics.

[bb0170] Wu S., Yang J., Dong H., Liu Q., Li X., Zeng X., Bai W. (2021). Key aroma compounds of Chinese dry-cured Spanish mackerel (*Scomberomorus niphonius*) and their potential metabolic mechanisms. Food Chemistry.

[bb0175] Yao Y., Fu B., Han D., Zhang Y., Liu H. (2020). Formate-dependent Acetogenic utilization of glucose by the fecal Acetogen *Clostridium bovifaecis*. Applied and Environmental Microbiology.

[bb0180] Ye L., Liu G., Yao T., Lu J. (2021). Monitoring of antimicrobial resistance genes in the spotted sea bass (*Lateolabrax maculatus*): Association with the microbiome and its environment in aquaculture ponds. Environmental Pollution.

[bb0185] Zagrodnik R., Łaniecki M. (2017). Hydrogen production from starch by co-culture of *Clostridium acetobutylicum* and *Rhodobacter sphaeroides* in one step hybrid dark- and photofermentation in repeated fed-batch reactor. Bioresource Technology.

